# Derivation of human retinal cell densities using high‐density, spatially localized optical coherence tomography data from the human retina

**DOI:** 10.1002/cne.25483

**Published:** 2023-04-19

**Authors:** Janelle Tong, Vincent Khou, Matt Trinh, David Alonso‐Caneiro, Barbara Zangerl, Michael Kalloniatis

**Affiliations:** ^1^ Centre for Eye Health University of New South Wales (UNSW) New South Wales Sydney Australia; ^2^ School of Optometry and Vision Science University of New South Wales (UNSW) New South Wales Sydney Australia; ^3^ School of Optometry and Vision Science Centre for Vision and Eye Research Contact Lens and Visual Optics Laboratory Queensland University of Technology Queensland Brisbane Australia; ^4^ School of Science, Technology and Engineering University of Sunshine Coast Queensland Sippy Downs Australia; ^5^ Coronary Care Unit Royal Prince Alfred Hospital New South Wales Sydney Australia; ^6^ Department of Optometry School of Medicine Deakin University Victoria Waurn Ponds Australia

## Abstract

This study sought to identify demographic variations in retinal thickness measurements from optical coherence tomography (OCT), to enable the calculation of cell density parameters across the neural layers of the healthy human macula. From macular OCTs (*n* = 247), ganglion cell (GCL), inner nuclear (INL), and inner segment–outer segment (ISOS) layer measurements were extracted using a customized high‐density grid. Variations with age, sex, ethnicity, and refractive error were assessed with multiple linear regression analyses, with age‐related distributions further assessed using hierarchical cluster analysis and regression models. Models were tested on a naïve healthy cohort (*n* = 40) with Mann–Whitney tests to determine generalizability. Quantitative cell density data were calculated from histological data from previous human studies. Eccentricity‐dependent variations in OCT retinal thickness closely resemble topographic cell density maps from human histological studies. Age was consistently identified as significantly impacting retinal thickness (*p* = .0006, .0007, and .003 for GCL, INL and ISOS), with gender affecting ISOS only (*p* < .0001). Regression models demonstrated that age‐related changes in the GCL and INL begin in the 30th decade and were linear for the ISOS. Model testing revealed significant differences in INL and ISOS thickness (*p* = .0008 and .0001; however, differences fell within the OCT's axial resolution. Qualitative comparisons show close alignment between OCT and histological cell densities when using unique, high‐resolution OCT data, and correction for demographics‐related variability. Overall, this study describes a process to calculate in vivo cell density from OCT for all neural layers of the human retina, providing a framework for basic science and clinical investigations.

## Graphical abstract

Pseudocolor cluster maps and regression models describing normal, age‐related change in the ganglion cell layer, inner plexiform layer, and inner segment–outer segment region of photoreceptors, as derived from optical coherence tomography data.

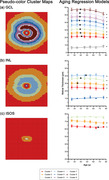

## 1 INTRODUCTION

A core principle underpinning vision science is that there should be a meaningful relationship between the cellular and physiological processes involved in the visual pathway and the perceptual output of vision (Barlow, 1972; Teller, 1984). As the first component of the visual pathway at which light is converted to neurochemical transmissions, retinal cell biology has been studied and characterized extensively, with alterations in retinal neuronal components attributed to a range of ocular pathologies and their associated functional deficits. For example, reductions in photoreceptor cell numbers amidst numerous other changes in the outer retinal layers have been observed in histological samples from human eyes with age‐related macular degeneration (Curcio et al., 1996), and in mouse models of retinitis pigmentosa apoptosis of photoreceptors and bipolar cells has been reported (Portera‐Cailliau et al., 1994; Zhang et al., 2010). Similarly, in human and primate histological studies of glaucoma, reduced retinal ganglion cell (GC) densities and optic nerve axon numbers have been correlated with reductions in visual field sensitivity (Antwi‐Boasiako et al., 2021; Garway‐Heath et al., 2000; Kerrigan‐Baumrind et al., 2000). However, these cellular components cannot be readily visualized in vivo with widely available imaging technologies, such that this knowledge of structural changes at the cellular level and quantitative relationships with outputs of vision cannot be directly translated to applications where cell parameters are not directly measured.

Retinal thickness measurements acquired in vivo using optical coherence tomography (OCT) are commonly applied in lieu of cellular parameters in both research investigations comparing structural and functional outputs and ophthalmic clinical care (Hood et al., 2019; Leite et al., 2012; Ly et al., 2018). In these applications, there is an underlying assumption that thinner retinal layers are analogous to a loss of corresponding retinal cells. However, quantitative comparisons between retinal layer measurements derived from OCT and histological cell parameters have been limited by available OCT measurements from commercial software, which are typically averaged over concentrically arranged subfields, for example, in the Early Treatment for Diabetic Retinopathy Study grid (Brandl et al., 2019; Cvenkel & Kontestabile, 2011). First, the sizes of individual subfields are relatively large, typically leading to few averaged retinal thickness measurements being calculated, and even fewer measurements along each meridian, limiting the number of measurements comparable to each histological slice. Moreover, such grids are based on the assumption that the distributions of retinal thickness measurements in healthy and pathological retinas occur in a symmetrical and concentric manner. However, studies using spatial cluster analysis of OCT measurements from healthy retinas have identified variably symmetric patterns of age‐related change in different retinal layers (Tong et al., 2019; Trinh et al., 2021; Yoshioka et al., 2017). The relative sparseness of obtained retinal thickness measurements and variable distributions of measurements for different retinal layers suggest that commercially available OCT measurement grids may be inadequate to enable the visualization of patterns of OCT‐derived retinal thickness measurements to compare to histological distributions. Furthermore, commercial OCT software calculate retinal thickness values axially (Alonso‐Caneiro et al., 2016), which do not consider alterations in retinal tilt with increasing eccentricity or tilted scan acquisition.

In the present study, a customized high‐density grid was applied to develop an OCT‐based model representing distributions of the layers of the macula corresponding to cellular structures, namely, the ganglion cell layer (GCL), inner nuclear layer (INL), and inner segment–outer segment layer (ISOS), in healthy human retinas. These models subsequently enabled comparisons between OCT data and density data for the corresponding cell bodies, as reported by previous studies using histological samples of the human retina (Curcio & Allen, 1990; Curcio et al., 1990; Masri et al., 2021), with the ISOS chosen to match the photoreceptor outer segment densities described in Curcio et al. (1990). Verification of whether OCT‐derived retinal thickness measurements can adequately describe corresponding retinal cellular changes is imperative to confirm the role of retinal thicknesses as a surrogate structural measure. Should OCT reflect histological cell density data, the subsequent ability to calculate in vivo cell densities would be a powerful tool for the detection of a variety of retinal pathologies and enabling in‐depth studies of the human retina.

## 2 MATERIALS AND METHODS

### 2.1 Participant recruitment and data collection

This study adhered to the tenets of the Declaration of Helsinki throughout its duration. Healthy participants were retrospectively recruited from patients attending Centre for Eye Health (Sydney, Australia) for comprehensive eye examinations, who had provided written informed consent for clinical data to be used for research purposes per ethics protocols approved by the University of New South Wales Australia Human Research Ethics Panel. Consistent with previous studies in which participant data from this study were used (Phu et al., 2020; Tong et al., 2019; Trinh et al., 2021; Yoshioka et al., 2017), participants were deemed suitable for inclusion if there was no optic nerve head pathology in both eyes and no macular pathology in at least one eye. Additional inclusion criteria included visual acuity better than 20/25 (logMAR < .1) for participants under 60 years old or 20/30 (logMAR < .2) for participants over 60 years old, intraocular pressure <22 mmHg in both eyes, mean spherical equivalent refraction between +3.00 and −6.00 diopters and astigmatism <3.00 diopters. Where one eye met inclusion criteria, this eye was selected for further analysis, while if both eyes were suitable one eye was selected at random; that is, only one eye was included per participant.

The study cohort was separated into two subcohorts to enable model development and testing to be performed without cohort duplication potentially inflating model performance (Table 1). Data from 247 participants (modeling cohort) ranging from 20 to 85 years of age were used for model development. Data from 40 participants (test cohort), aged between 20–25 and 60–65 years of age, were used for subsequent model testing, with the specific age ranges chosen to be outside those used in previous histological studies (Curcio & Allen, 1990; Curcio et al., 1990; Masri et al., 2021).

**TABLE 1:** Demographic characteristics of the cohorts included in the present study.

	Modeling cohort			
	All	GCL and ISOS age‐similar	INL age‐similar	Test cohort
**Number of eyes**	247	36	139	40
**Age, y (mean ± *SD*, range)**	50.3 ± 14.5, 20.2–84.9	32.4 ± 3.7, 27.1–39.2	46.4 ± 7.2, 30.1–57.0	42.7 ± 19.9, 20–66
***Rx*, *D* (mean ± *SD*)**	−.51 ± 1.75	−1.59 ± 1.79	−.70 ± 1.56	−.87 ± 1.86
**Sex (M:F)**	141:106	27:11	87:53	20:20
**Eye included (OD:OS)**	129:118	21:17	81:59	20:20
**Ethnicity (White:Asian)**	156:91	18:20	82:58	17:23
**BCVA logMAR (mean ± *SD*)**	.004 ± .10	−.04 ± .09	−.02 ± .08	.0065 ± .06

*Note*: Within the modeling cohort, all eyes were used to develop normative models, while the age‐similar cohorts were chosen as similar to historical histological cohorts (Curcio & Allen, 1990; Curcio et al., 1990; Masri et al., 2021), for use as comparisons to corrected data from the test cohort and derivation of cell densities from histological data. See Table S1 for demographic characteristics of histological cohorts.

Abbreviations: BCVA, best‐corrected visual acuity; *D*, diopters; F, female; GCL, ganglion cell layer, logMAR, log minimum angle of resolution; INL, inner nuclear layer; ISOS, inner segment–outer segment; M, male; OD, right eye; OS, left eye; *Rx*, refractive error; *SD*, standard deviation; y, years.

**FIGURE 1:**
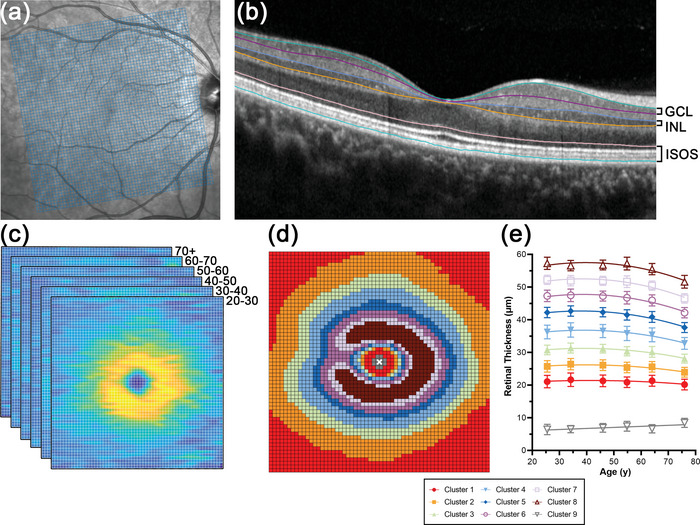
Schematics describing retinal thickness extraction and analyses performed for normative model development: (a) The 60 × 60 grid over which retinal thickness measurements were extracted, spanning a total area of 6880 μm × 6880 μm. As depicted, the grid was centered on the foveal center and tilt was adjusted according to fovea to optic disc tilt. (b) Macular optical coherence tomography (OCT) B‐scan delineating the boundaries of the ganglion cell layer (GCL), inner nuclear layer (INL), and inner segment–outer segment layer (ISOS). (c) After extraction, retinal thickness measurements for each grid square were averaged by decade cohorts, after which hierarchical cluster analysis was performed. (d) A pseudocolor theme map of GCL thickness derived from cluster analysis, where grid squares colored the same show statistically similar age‐related change. (e) Regression analyses depicting rates of age‐related change in GCL thickness. Each regression line is colored to match locations pooled in each cluster, as depicted in (d).

OCT posterior pole scans acquired with the Spectralis SD‐OCT (Heidelberg Engineering, Heidelberg, Germany) during participants’ clinical examinations were processed for eyes flagged for inclusion in further analyses. The preset posterior pole scan under the Spectralis Glaucoma Module consists of 61 horizonal B‐scans spaced 120 μm apart, covering a total retinal area of 8600 μm × 7167 μm, and the Automated Real Time function set to 9 to improve image quality. The angle between the foveal and optic disc centers was automatically computed by the HRA/Spectralis Viewing Module (version 6.9.5.0, Heidelberg Engineering, Heidelberg, Germany), aided by automated detection of the fovea and optic disc margins during scan acquisition, which was manually inspected and corrected as required. For individual B‐scans, locations impacted by significant anatomical artifacts, such as the optic nerve head or intraretinal blood vessels, or a signal strength <15 dB were excluded. Automated segmentation of the GCL, INL, and ISOS boundaries (between the external limiting membrane and the retinal pigmented epithelium) was manually reviewed and corrected as required by two researchers experienced in retinal layer segmentation (VK and MT, Figure 1), with agreement between them constituting the “ground‐truth” segmentation (Tian et al., 2016; Trinh et al., 2021).

### 2.2 Experimental design and statistical analysis

For extraction of OCT‐derived thickness measurements from the GCL, INL, and ISOS region of photoreceptors at a higher spatial resolution than available in commercial OCT review software with adjustment for OCT B‐scan tilt, a custom algorithm was written using MATLAB Version 9.6 (MathWorks, Natick, MA, USA). The fundamental components of the algorithm have been described previously (Tong et al., 2020). In short, after data were extracted in a RAW format, B‐scan tilt was determined by calculating the tangent to the retinal pigment epithelium boundary along each location. From the perpendicular to each tangent, the pixel distance between the intersections for the inner and outer boundaries of the GCL, INL, and ISOS were calculated and multiplied by the axial pixel resolution of the Spectralis OCT, 3.87 μm, to calculate tilt‐corrected retinal thicknesses.

Subsequently, retinal thickness measurements were extracted over a 6880 μm × 6880 μm area centered on the foveal center, per the standard 8 × 8 grid available on the HRA/Spectralis Viewing Module. An additional benefit of the smaller extraction area relative to scan acquisition area is limited data loss secondary to poor segmentation at the scan acquisition area border. OCT data were extracted at a sampling density of 60 × 60 grid squares, with measurements averaged across each grid square measuring 114.67 μm × 114.67 μm, providing a 56.25‐fold higher sampling density than the standard 8 × 8 grid. To facilitate data analysis across participants, all data were converted to right eye format after extraction.

Statistical analyses enabling model development and testing were conducted using SPSS Statistics Version 23.0 (IBM Corporation, New York, NY, USA) and GraphPad Prism Version 8.4.3 (GraphPad, La Jolla, CA, USA). Age, mean spherical equivalent refractive error, sex, ethnicity, and best corrected visual acuity were extracted from clinical records. Age was a demographic variable of particular interest, given previous reports of age‐related decline in both OCT‐derived retinal thickness measurements and histological cell densities (Aggrawal et al., 2007; Gao & Hollyfield, 1992; Gartner & Henkind, 1981; Harman et al., 2000; Mwanza et al., 2011; Tong et al., 2019; Trinh et al., 2021; Yoshioka et al., 2017); given the relatively limited age ranges included in histological studies, derivation of age‐correction factors would be optimal for a broader application of histological data to cohorts that are not necessarily similar in age. To ensure a complete investigation of other demographic variables that may impact retinal thickness measurements, for each of the GCL, INL, and ISOS, measurements across the entire 60 × 60 grid were averaged for each participant in the modeling cohort, and multiple linear regression was conducted with retinal thickness measurements set as the dependent variable and the above demographic variables set as the main effects. Should age demonstrate a significant relationship with a retinal thickness measurement, the age‐coefficients derived from multiple linear regression and standard linear regression, which is only between age and the retinal thickness measurement in question, were compared. Differences in coefficients exceeding 10% indicated meaningful contribution of the other tested demographic variables in the multiple linear regression models (Budtz‐Jorgensen et al., 2007). Subsequently, corrected coefficients for significant variables other than age were calculated using multiple linear regression repeated with nonsignificant demographic variables removed, akin to backward stepwise elimination (Derksen & Keselman, 1992).

Given previous reports of nonlinear patterns of age‐related decline in various retinal layers, with different rates of change dependent on macular location (Mwanza et al., 2011; Tong et al., 2019; Trinh et al., 2021; Yoshioka et al., 2017), hierarchical cluster analysis was applied to identify retinal locations demonstrating statistically similar aging characteristics irrespective of outcomes of multiple linear regression analysis. Cluster analysis has been previously applied to characterize distributions of amino acid signatures in retinal neurons in the normal and degenerating vertebrate retina (Jones et al., 2003; Kalloniatis et al., 1996; Marc & Jones, 2002; Marc et al., 1995); in this study, a similar approach was applied to enable the development of regression models describing change in retinal thickness measurements as a function of age (Tong et al., 2019; Trinh et al., 2021; Yoshioka et al., 2017). Participants in the modeling cohort were grouped by decade brackets (20 to <30 years, 30 to <40 years, etc.), with participants 70 years of age and more grouped together due to relatively small sample size in this subcohort. For the relevant retinal layers, for each grid square measurements were averaged across each age bracket prior to hierarchical cluster analysis using within‐groups linkage and squared Euclidean distance. Cluster separability was determined by calculating *d*′ for each cluster pair, based on the mean (*x*) and standard deviation (*σ*) retinal thickness measurement for each cluster:

$$d^{\prime }\;\;=\;\;\frac{|x_{1}-x_{2}|}{\sqrt{.5\times \left(\sigma_{1}^{\;2}+\sigma_{2}^{\;2}\right)}}$$

(Phu et al., 2017; Tong et al., 2019)

The *d*’ threshold criterion was set at 1, equivalent to 1 *SD* separation between clusters. Given the axial resolution of the Spectralis OCT is 3.87 μm, this was applied as an additional criterion to ensure at least 1 pixel's difference between cluster means. Should *d*′ < 1 or the difference in means be less than 3.87 μm for a particular cluster pair, these clusters were deemed not statistically separable and pooled together. Subsequently, retinal thickness measurements were pooled by cluster and age bracket, and quadratic and linear regression models were applied, with extra sums‐of‐squares *F*‐test comparisons performed to identify the most appropriate regression model describing aging change for that cluster and retinal layer. Where a quadratic linear regression model was preferred, the point of inflection (*x*), or the point at which retinal thickness begins to decline, was calculated from quadratic (*a*) and linear (*b*) coefficients of the regression model:

$$x\;=\;-\frac{b}{2a}$$

(Tong et al., 2019)

To enable comparisons of rate of change described by these models between clusters and between retinal layers, the average annual percentage change was calculated from peak retinal thicknesses predicted by the quadratic and linear regression models.

Testing the derived age‐correction factors on a naïve cohort would verify the generalizability of the regression models and in turn of parameters derived from the histological data sets. From the modeling cohort, participants falling within the age range of each histological cohort were selected as age‐similar subcohorts (Tables 1 and 2); that is, all participants aged between 27 and 40 years were chosen as GCL and ISOS age‐similar subcohorts, similar to the age ranges of the Curcio and Allen (1990) for GC data and Curcio et al. (1990) for photoreceptor data, and all participants aged between 30 and 57 years were chosen as the INL age‐similar subcohort to match the age range of the Masri et al. (2021) INL cohort. Although this resulted in a slightly younger and older mean age in the GCL/ISOS and INL age‐similar cohorts respectively, and a greater proportion on male participants in these modeling cohorts relative to the original histological cohorts, this was performed over preselection of groups matching the mean ages of the histological cohorts exactly to minimize selection bias. Meanwhile, the GCL, INL, and ISOS thickness measurements from the test cohort were age‐corrected to 32.4, 46.4, and 32.4 years, respectively, matching the mean ages of the age‐similar subcohorts, with the appropriate age‐correction factor applied depending on the cluster in which each retinal location fell. Correction for other significant demographic variables identified through multiple linear regression analyses, to match age‐similar subcohort characteristics, was also performed as required. Hodges–Lehmann differences in median age‐similar and corrected GCL, INL, and ISOS thickness measurements across the 60 × 60 measurement grid were computed with unpaired Mann–Whitney tests, and differences as a function of retinal thickness were visualized with Bland–Altman plots. Linear regression models were also applied with computation of 95% prediction intervals to Bland–Altman plots to determine whether biases altered with varying retinal thickness, and extra sums‐of‐squares *F* tests were used to determine significance of regression models. Maximum predicted differences between age‐similar and age‐corrected data were calculated by adding half the prediction interval width to the maximum absolute difference predicted by the linear models. Finally, test cohort data were used to plot differences in tilt‐adjusted and uncorrected GCL, INL, and ISOS thickness as a function of B‐scan tilt, with appropriate regression analyses applied to enable the visualization of alterations in retinal thickness with increasing tilt. Across all analyses, the threshold for statistical significance was set at *p* < .05.

**TABLE 2:** Demographic characteristics of histological cohorts from previously reported data (Curcio & Allen, 1990; Curcio et al., 1990; Masri et al., 2021).

	GC density (Curcio & Allen, 1990)	Ph density (Curcio et al., 1990)	INL cell density (Masri et al., 2021)
**Number of eyes**	6	8	6
**Age, y (mean ± *SD*, range)**	34.0 ± 3.6, 27.0–37.0	34.8 ± 4.7, 27.0–44.0	44.2 ± 9.8, 31.0–56.0
**Sex (M:F)**	3:3	3:5	1:5
**Eye included (OD:OS)**	3:3	3:5	3:3

Abbreviations: F, female; GC, ganglion cell; INL, inner nuclear layer; M, male; N/A, not available; OD, right eye; OS, left eye; Ph, photoreceptor; *SD*, standard deviation; y, years.

### 2.3 Linking OCT data to historical histological cohorts

Direct comparisons of OCT and histological data would enable the estimation of retinal cell density, and subsequently retinal cell counts, from corresponding retinal layer thickness measurements derived from OCT. Histological GC data were derived from Curcio and Allen (1990), who reported GC density in cells per en face square millimeter (cells/mm^2^) as a function of eccentricity along each principal meridian. For the derivation of GC density per the 60 × 60 measurement grid, similar to previous studies (Garway‐Heath et al., 2000; Raza & Hood, 2015; Yoshioka et al., 2018), GC densities between the principal meridians were linearly interpolated at 5 μm intervals. GC densities were subsequently averaged across each grid square area. For the subsequent application of OCT data, GC density in cells per cubed millimeter (cells/mm^3^) was calculated by dividing the GC density in cells/mm^2^ by the mean GCL thickness for the corresponding grid square in the age‐similar subcohort.

Histological INL data were obtained from Masri et al. (2021), who reported sum‐of‐exponential equations describing cell density for various cell populations stratifying to different layers within the INL as a function of eccentricity. These equations were used to calculate cell densities at 5 μm intervals for each cell type residing within the INL; at each eccentricity, glycinergic and GABAergic amacrine cell densities were summed to derive total amacrine cell density, and OFF midget bipolar, ON bipolar, DB3a, and DB3b cell densities were summed to derive total bipolar cell density. These were then summed with total horizontal and Müller cell densities to obtain total DAPI‐stained cell density, and given that 10.6% of INL cells remain unlabeled by DAPI (Masri et al., 2021), this correction factor was applied to determine the density of all cells within the INL in cells/mm^2^. Relative proportions of total amacrine, total bipolar cell, total horizontal cell, and Müller cell densities were calculated from total INL cell densities. Masri et al. (2021) only described INL cell densities along the temporal meridian; in the absence of meridian‐specific cell density data, it could only be assumed that INL cell density only varied as a function of eccentricity. As such, INL cell densities were divided by INL thickness values along the temporal meridian only to derive volumetric INL cell densities and extrapolated across all angular eccentricities within the 60 × 60 measurement grid. These were then multiplied by INL thickness measurements and the relative proportions of total amacrine, total bipolar cell, total horizontal cell, and Müller cell densities to derive potential cell densities of individual cell populations in cells/mm^2^.

Lastly, histological photoreceptor data were obtained from Curcio et al. (1990), who reported cone and rod inner segment density in cells/mm^2^ as a function of eccentricity along each principal meridian. However, due to the co‐stratification of cone and rod inner segments, the corresponding ISOS represents both cell classes, with varying proportions along the en face plane with increasing eccentricity. As such, the relative contribution of cone and rod inner segments to ISOS thickness would depend on cone and rod diameter, which themselves vary with eccentricity (Curcio et al., 1990; Jonas et al., 1992; Scoles et al., 2014). Outside of the rod‐free zones, reported as between 126 and 200 μm and between different principal meridians (Curcio et al., 1990), cone diameter as a function of eccentricity was determined using Akima spline interpolation for each principal meridian from data reported by Scoles et al. (2014). Assuming maximum packing with hexagonal inner segment cross sections, the relative en face area covered by cone inner segments was calculated using corresponding cone density data at 5 μm eccentricity intervals for each principal meridian. The remainder of the surface area was assumed to be covered by rod inner segments, and once again assuming hexagonal inner segment cross sections, rod diameter was calculated based on rod density data. However, as rods are relatively sparse at the central retina, calculations at central locations may have resulted in overestimation of rod diameter, and as such linear regression models were fit through the data. From estimated rod diameters, the ratio of rod to cone inner segment areas was used as a correction factor applied to rod density data and enabled the calculation of equivalent cone density as a function of eccentricity per principal meridian. As per GC analyses, equivalent cone density was linearly interpolated between principal meridians and averaged across 60 × 60 grid square locations, and subsequently divided by ISOS thickness measurements from the age‐similar subcohort to calculate equivalent cone density in cells/mm^3^.

## 3 RESULTS

### 3.1 Normative models of GCL, INL, and ISOS thickness

Normative model development involved the identification of demographic values potentially influencing neural retinal layer thickness and characterizing variations with eccentricity, enabling comparison to histological topographic maps. For global GCL and INL measurements averaged across the whole macula, multiple linear regression analyses indicated that age significantly influenced retinal thickness measurements (*p* = .0004–.006, Table 3). Additionally, significant coefficients for refractive error and sex were observed for the INL and ISOS, respectively (*p* = .009 and .0001 respectively). However, the difference between age coefficients derived from multiple and standard linear regression analyses for the GCL was only 3.49%, indicating that correcting for refractive error would not produce meaningful differences in observed aging trends in the INL. Conversely, the 10.94% difference in age coefficients in the INL‐suggested correction for refractive error and sex would be required to derive accurate models of age‐related change in this retinal layer, with a corrected parameter estimate of .201 for every diopter of refractive error and .594 for sex after multiple regression models were repeated with the removal of nonsignificant variables. Meanwhile, no significant parameters were observed for global ISOS measurements, suggesting that averaged ISOS thicknesses across the whole macula do not vary with the studied demographic factors.

**TABLE 3:** Multiple linear regression analyses between demographic variables and ganglion cell layer (GCL), inner nuclear layer (INL), and inner segment–outer segment (ISOS) thickness measurements.

Multiple linear regression	GCL		INL		ISOS	
Variables	Parameter estimate ± *SE*	*p* Value	Parameter estimate ± *SE*	*p* Value	Parameter estimate ± *SE*	*p* Value
**Age**	−.044 ± .012	.0004	−.036 ± .010	.0006	−.040 ± .025	.10
***Rx***	.20 ± .093	.03	.237 ± .079	.003	.147 ± .19	.43
Corrected estimate	–		.201 ± .076		–	
**Sex**	.192 ± .305	.53	.543 ± .258	.04	.864 ± .613	.16
Corrected estimate	–		.594 ± .253		–	
**Ethnicity**	.447 ± .332	.18	.420 ± .281	.14	.435 ± .667	.52
**BCVA**	−2.24 ± 1.75	.18	−1.44 ± 1.48	.33	−.139 ± 3.52	.97
**Standard linear regression**						
**Age**	−.046 ± .01	<.0001	−.032 ± .009	.0001	–	–
**Difference (%)**	−3.49		10.94		–	

*Note*: As age was a significant variable in all analyses, comparisons between age‐coefficients derived from multiple and standard linear regression were performed. The corrected estimate for sex and refractive error and INL was derived from multiple linear regression with the removal of nonsignificant variables, akin to backward stepwise elimination. As no significant parameters from multiple linear regression were noted for ISOS, comparison with linear regression was not performed.

Abbreviations: BCVA, best corrected visual acuity; *Rx*, refractive error; *SE*, standard error.

**FIGURE 2:**
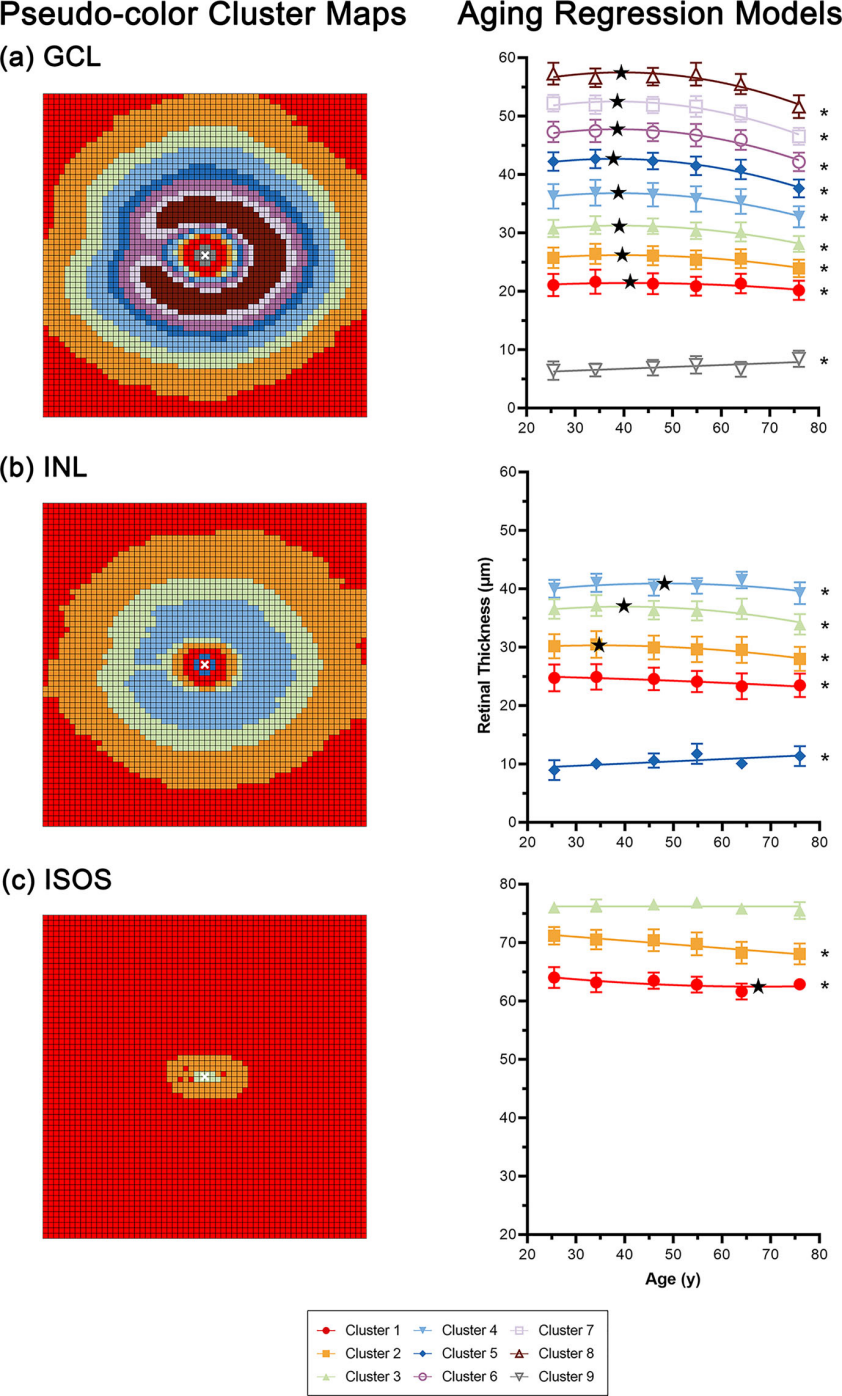
Pseudocolor cluster maps (left column) and regression models describing age‐related change (right column) in the (a) ganglion cell layer (GCL), (b) inner nuclear layer (INL), and (c) inner segment–outer segment layer (ISOS). In both, locations that are colored the same indicate those that show statistically similar changes with age, with 9, 5, and 3 statistically separable clusters identified for the GCL, INL, and ISOS, respectively. In regression models, data were pooled per the clustered locations in pseudocolor maps and per decade bracket, with mean and standard deviation for each data point depicted. Black stars indicate points of inflection for clusters where quadratic regression models were preferred, and asterisks indicate significant age‐regression models. See Table 4 for coefficients describing regression models.

Hierarchical cluster analyses of the GCL, INL, and ISOS were conducted to identify macular locations that demonstrated similar change with age, and that would therefore be suitable to pool together in subsequent regression analyses. Although multiple linear regression analyses did not suggest significant changes in ISOS thickness with age, analyses were still performed as global ISOS measurements averaged across the entire macula may mask location‐specific age‐related changes. Hierarchical cluster analyses revealed nine statistically separable clusters in the GCL, five clusters in the INL, and three clusters in the ISOS (Figure 2). In the GCL and INL, these clusters demonstrated a concentric, horseshoe appearance indicating asymmetry in retinal thickness measurements along the horizontal axis, closely resembling patterns of eccentricity‐dependent variation previously described in histological studies (Curcio & Allen, 1990). While the mean GCL thickness at the center‐most cluster was 7.07 μm, this is likely an artifact of segmentation of the RNFL–GCL and GCL–INL boundaries, as GCs are absent at the foveola (Curcio & Allen, 1990). Of interest is the horseshoe pattern in both GCL and INL profiles, and the peaked ISOS thickness at the foveal center with steep reduction from the parafovea to mid‐peripheral locations; these patterns of change closely resemble topographic cell density distributions as described in histological studies (Curcio & Allen, 1990; Curcio et al., 1990). These similarities in eccentricity‐dependent variations provided support for the linkage of high density OCT measurements and histological cell density data.

Subsequent regression models pooling data per cluster and decade bracket revealed across the GCL, INL, and ISOS, quadratic models best described age‐related change at all locations except for subfoveal locations, where a linear model was preferred (*p* = .10–.26 at the central macula, *p* ≤ .0001–.01 otherwise, Table 4). Moreover, slope parameter analyses indicated that while the central‐most cluster of the ISOS did not demonstrate notable change in ISOS thickness with age (*p* = .14), other locations did demonstrate a significant decline with age (*p* ≤ .0001 for both), suggesting that the results of multiple linear regression analysis were primarily influenced by foveal ISOS measurements. Points of inflection derived for quadratic regression models indicated that decline in GCL thickness began between 37.8 and 41.3 years across the macula. Similar trends were noted in the INL, with points of inflection between 34.8 and 48.0 years across the macula, while the majority of the ISOS displayed a linear rate of change with age. Across the macula, the annual percentage rate of decline was calculated as −.34% for the GCL, −.19% for the INL, and −.07% for the ISOS, which was reasonably consistent across all locations, excluding the central fovea, where more variable rates of decline were observed (Table 4).

**TABLE 4:** Coefficients of regression models describing age‐related change in the ganglion cell layer (GCL), inner nuclear layer (INL), and inner segment–outer segment (ISOS).

	*a*	*b*	*c*	*p* Value	*x* (y)	Annual rate of change (%)
**GCL**						
Cluster 1	−.0010	.08	19.80	<.0001	41.3	−.19
Cluster 2	−.0017	.13	23.58	<.0001	39.7	−.29
Cluster 3	−.0023	.18	27.79	<.0001	39.1	−.33
Cluster 4	−.0028	.22	32.51	<.0001	38.9	−.36
Cluster 5	−.0034	.25	37.88	<.0001	37.8	−.37
Cluster 6	−.0038	.29	42.06	<.0001	38.7	−.37
Cluster 7	−.0040	.31	46.56	<.0001	38.5	−.36
Cluster 8	−.0041	.33	51.02	<.0001	39.5	−.33
Cluster 9	N/A	.03	5.46	.26	N/A	.58
**INL**						
Cluster 1	−.0002	−.01	25.35	.01	−25.5	−.12
Cluster 2	−.0013	.09	28.74	<.0001	34.8	−.22
Cluster 3	−.0021	.16	33.67	<.0001	39.8	−.25
Cluster 4	−.0016	.16	37.14	<.0001	48.0	−.15
Cluster 5	N/A	.04	8.56	.11	N/A	.44
**ISOS**						
Cluster 1	.00089	−.12	66.55	<.0001	67.5	−.07
Cluster 2	N/A	−.06	72.95	<.0001	N/A	−.09
Cluster 3	N/A	−.01	76.77	.14	N/A	−.01

*Note*: Cluster numbers are labeled per Figure 2, where lower numbers indicate relatively peripheral locations. *p* Values indicate outcomes of *F*‐test between quadratic and linear regression models, with a significant result indicating quadratic models best fit the data, and points of inflection (*x*) were derived for quadratic regression models. *p* Values of N/A indicate clusters where nonsignificant changes with age were detected on linear regression, and therefore comparisons between quadratic and linear regression were not performed. Annual rates of change were then calculated as a percentage of peak retinal thickness predicted from the quadratic or linear models as appropriate. *a*, quadratic regression coefficient; *b*, linear regression coefficient; *c*, constant.

Abbreviation: y, years.

### 3.2 Correction using the derived normative models

**FIGURE 3:**
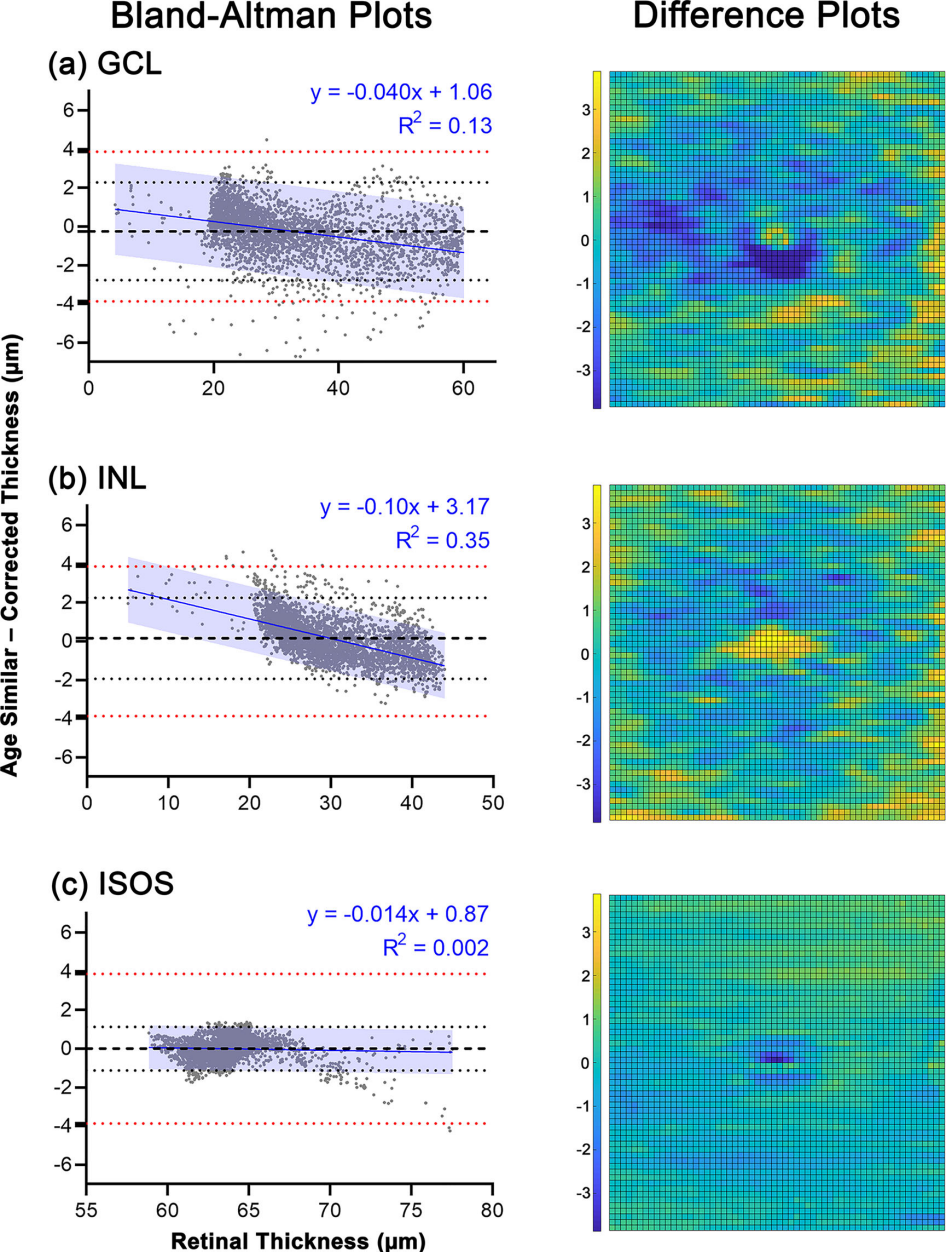
Bland–Altman plots (left column) and colored location‐specific difference plots (right column) describing differences in age‐similar and corrected (a) ganglion cell layer (GCL), (b) inner nuclear layer (INL), and (c) inner segment–outer segment layer (ISOS) thickness. In Bland–Altman plots, bias (black dashed lines) and 95% limits of agreement (black dotted lines) relative to the axial pixel resolution of the Spectralis OCT, 3.87 μm (red dotted lines), are shown. Linear regression models through the Bland–Altman plots (blue solid lines) and corresponding 95% prediction intervals (blue shading) are also shown, with the parameters of the linear models shown in blue text.

The normative models describing variations in GCL, INL, and ISOS thickness with demographic features were tested to determine generalizability of these models on other cohorts. With data in the test cohort corrected to a 32.4 year age‐equivalent for the GCL and ISOS and to a 46.4 year age‐equivalent for the INL, and adjusted for sex and refractive error in INL analyses, comparisons with uncorrected data from age‐similar participants in the modeling cohort (chosen to match the respective histological cohorts as closely as possible) revealed no significant differences in GCL and ISOS thicknesses (*p* = .85 and .28 respectively), but significantly thinner median INL in the corrected cohort (*p* = .03, Table 5). However, Bland–Altman comparisons revealed no notable bias with 95% limits of agreement falling well within the axial pixel resolution of the Spectralis OCT of 3.87 μm (Figure 3 and Table 5). While linear regression models were significant across all retinal layers (*p* < .000–.0024), low coefficients of determination (*R* ^2^) indicated relatively poor fits of the linear models to the data. Moreover, considering the 95% prediction intervals, within which 95% of data points are expected to fall, the maximum predicted differences between age‐corrected and age‐similar thicknesses were 3.71 μm for the GCL, 4.36 μm for the INL, and 1.32 μm for the ISOS. That is, all maximum differences fell within the axial pixel resolution of the Spectralis OCT for the GCL and ISOS, and within the equivalent of 2 pixels difference for the INL. Moreover, plots depicting differences per macular location revealed the largest absolute changes occurred around the central fovea, which is prone to inter‐individual differences owing to variations in foveal pit contour (Dubis et al., 2009) (Figure 3). As such, from a practical perspective, the differences in corrected and age‐similar thickness measurements can overall be considered to be insignificant.

**TABLE 5:** Differences in ganglion cell layer (GCL), inner nuclear layer (INL), and inner segment–outer segment (ISOS) thicknesses between the age‐corrected cohort and age‐similar participants derived from the modeling cohort, via Hodges–Lehmann differences in median thicknesses, Bland–Altman analyses, and subsequent linear regression analyses to Bland–Altman data.

	GCL	INL	ISOS
**Difference age‐corrected—age‐similar, μm (median, 95% CI)**	−.04, −.43 to –.35	−.29, −.55 to −.03	−.04, −.12 to –.04
***p* Value**	.85	.03	.28
**Bland–Altman bias, μm**	−.25	.15	.0008
**Bland–Altman 95% LOA, μm**	−2.78 to 2.28	−1.94 to 2.25	−1.13 to 1.13
**Linear regression**			
**Slope**	−.040	−.10	−.014
**Intercept**	1.06	3.17	.87
***R* ^2^**	.13	.35	.002
**95% prediction interval width, μm**	4.72	3.37	2.25

Abbreviation: LOA, limits of agreement.

### 3.3 Tilt‐adjustment of retinal thickness measurements

**FIGURE 4:**
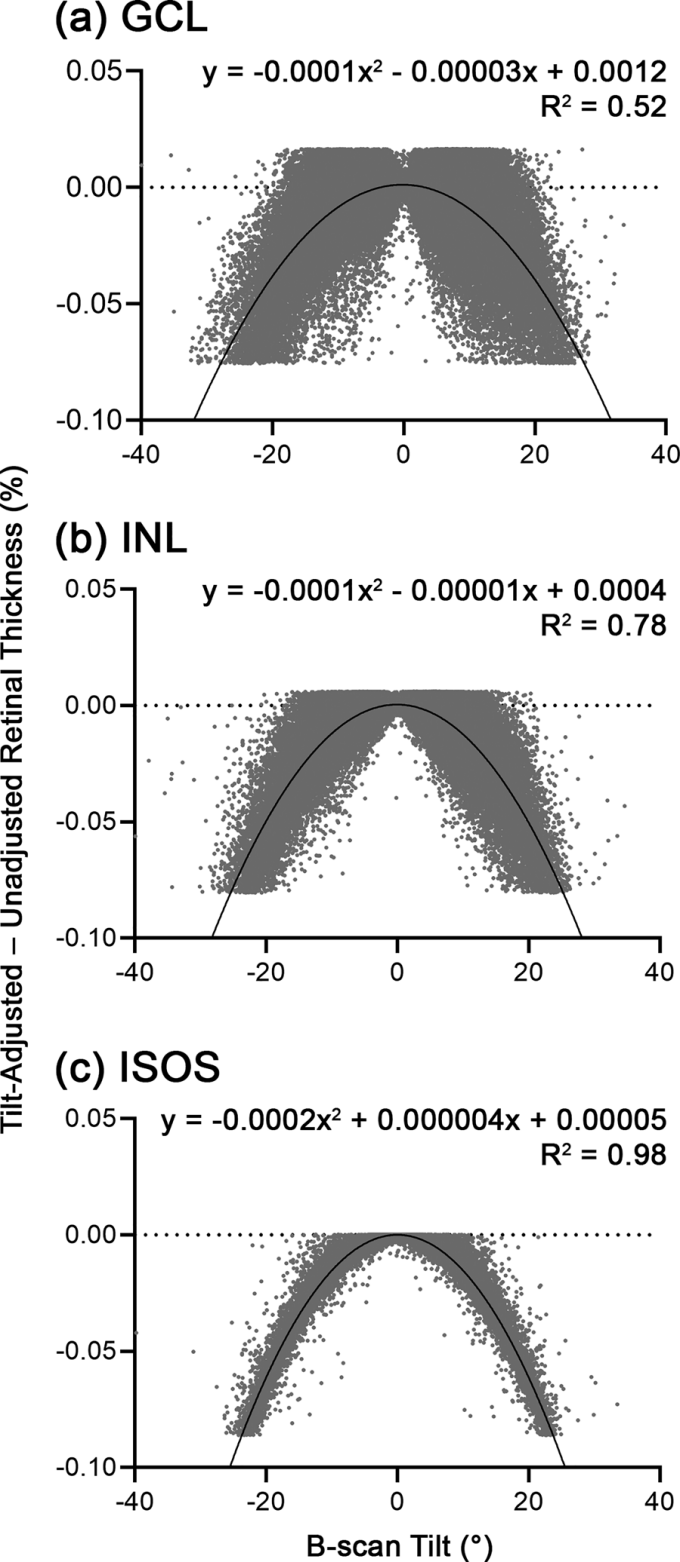
Percentage differences between tilt‐adjusted and unadjusted retinal thickness as a function of B‐scan tilt in the (a) ganglion cell layer (GCL), (b) inner nuclear layer (INL), and (c) inner segment–outer segment (ISOS) layer. The quadratic functions fit through the data and corresponding equations and coefficients of determination (*R* ^2^) are also shown.

While commercial OCT software compute and display axial retinal thickness measurements, by not considering B‐scan tilt there may be resultant systematic errors in the derived retinal thickness measurements (Alonso‐Caneiro et al., 2016; Uji et al., 2017). Analyses of percentage differences between tilt‐adjusted and unadjusted retinal thickness as a function of B‐scan tilt revealed notable differences with increasing B‐scan tilt across the GCL, INL, and ISOS (Figure 4), and quadratic regression models fitted through these data predicted a >5% difference at B‐scan tilts of ±22.61° for the GCL, ±20.17° for the INL and 18.10° for the ISOS. As maximum B‐scan tilts approached 40° in our data set of macular scans, these results suggest that corrections to obtain retinal thickness measurements perpendicular to the B‐scan tilt should be considered even for macular scans.

### 3.4 Cell density derivations from histological data

**FIGURE 5:**
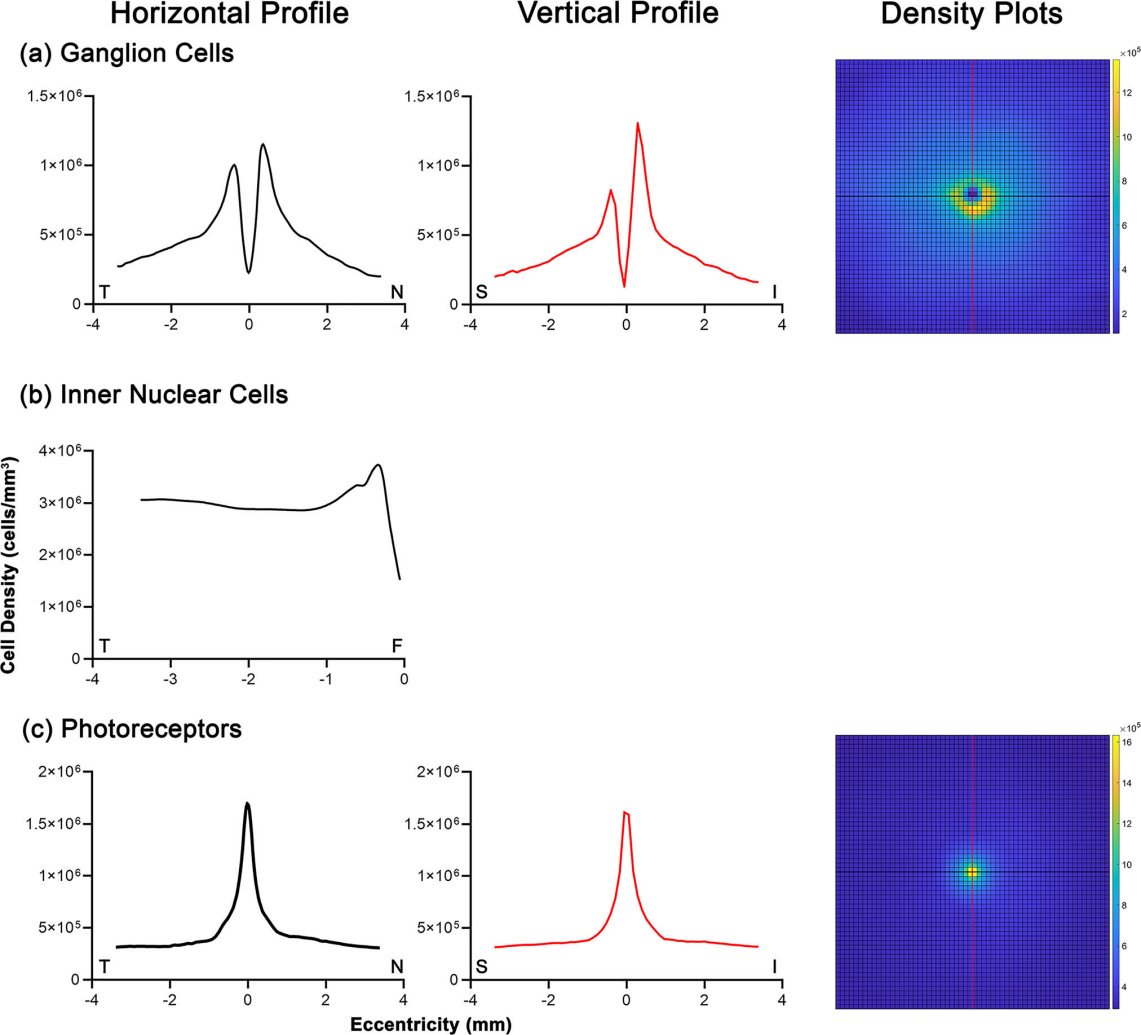
Volumetric cell densities (in cells/mm^3^) calculated from histological data and age‐similar optical coherence tomography (OCT) data for the corresponding retinal layer in (a) ganglion cells, (b) total inner nuclear layer cells, and (c) photoreceptors across the 60 × 60 grid (left column). Profiles of cell densities along the central horizontal meridian (black line, middle column) and vertical meridian (red line, right column) are also displayed, where T, N, S, and I denote temporal, nasal, superior, and inferior locations respectively. For the inner nuclear layer (INL), as cell density data were only available along the temporal meridian, volumetric cell density was calculated for this meridian then extrapolated to all other angular eccentricities, resulting in the symmetrical pattern observed. See Supporting Dataset 1 for numerical density measurements across the 60 × 60 grid.

**FIGURE 6:**
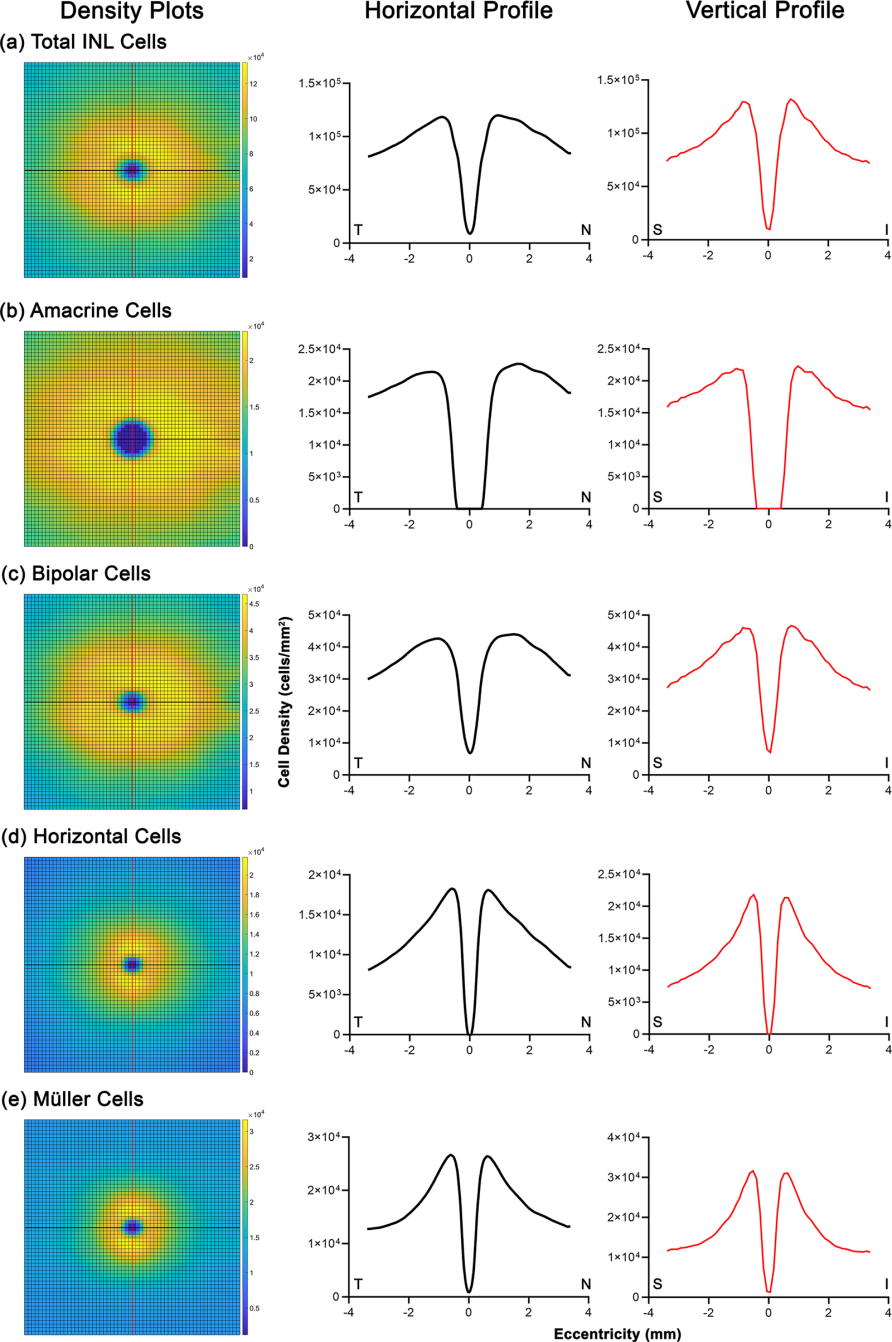
Areametric cell densities (in cells/mm^2^) calculated for (a) the total inner nuclear layer (INL), derived from volumetric cell density calculated for the temporal meridian then extrapolated to all other angular eccentricities divided by INL thickness across the 60 × 60 grid (left column). Areametric cell densities were subsequently calculated for individual subcomponents, that is, (b) amacrine, (c) bipolar, (d) horizontal, and (e) Müller cells. Profiles of cell densities along the central horizontal meridian (black line, middle column) and vertical meridian (red line, right column) are also displayed, where T, N, S, and I denote temporal, nasal, superior, and inferior locations, respectively. See Supporting Dataset 1 for numerical density measurements across the 60 × 60 grid.

After verifying the suitability of correction using the normative models to sufficiently minimize interindividual variability, derivations of volumetric cell density factors were performed across the 60 × 60 grid using OCT data from the age‐similar cohorts (varying slightly in mean age and sex distributions relative to corresponding histological cohorts). This was performed to enable the prediction of cell density and numbers from individuals’ OCT measurements (Figure 5, Supporting Dataset 1). While relatively concentric distributions were observed in photoreceptor densities, consistent with Curcio et al. (1990), asymmetries in peak and eccentricity‐dependent volumetric cell densities were noted in GC data. Along the horizontal axis, peak volumetric densities were observed at .35 mm from the foveal center nasally and .38 mm temporally, with nasal volumetric cell density exceeded temporal density until an eccentricity of 1.38 mm. Meanwhile, along the vertical axis, peak cell density was noted at .33 mm inferiorly and .38 mm superiorly, with inferior volumetric cell density, exceeded superior density until an eccentricity of .93 mm. These appear to reflect asymmetries in areametric GC densities between meridians, especially within .5 mm of the foveal center as depicted in Curcio and Allen (1990), particularly in the absence of notable asymmetry in GCL thickness along the vertical meridian (Figure 2). As INL cell densities were only available for the temporal meridian, under the assumption of consistent cell densities regardless of angular eccentricity a symmetrical, concentric pattern of volumetric cell density was obtained. However, subsequent derivation of areametric densities of calculated individual INL cell components revealed subtle asymmetries particularly along the horizontal axes (Figure 6, Supporting Dataset 1), consistent with asymmetry between nasal and temporal INL thickness (Figure 2).

## 4 DISCUSSION

Spatial patterns of eccentricity‐dependent change in GCL, INL, and ISOS thickness, as revealed by cluster analysis, closely resembled variations in GC and photoreceptor cell density described in histological studies. Combined with demonstrated reductions in inter‐cohort variability using the resultant normative models, these findings provide support for estimates of cell density from high spatial density OCT measurements as an appropriate surrogate to histologically measured cell densities. Changes in retinal thickness measurements derived from OCT have been presumed to correlate with a reduction in cell density, and while calculations of GC numbers from OCT have been previously described (Raza & Hood, 2015; Yoshioka et al., 2018), similar concepts had not been explored for other neural components of the retina. The cell density parameters presented in this study allow for the calculation of densities and numbers of GCs, cells residing in the INL or photoreceptors for a given eccentricity, area, and retinal thickness measurement, enabling readily accessible cell parameters to be applied in various settings.

### 4.1 Age‐related changes in retinal thickness

While histological studies have assessed age‐related changes in cell density to some extent (Curcio et al., 1993; Gao & Hollyfield, 1992; Harman et al., 2000), given the resource‐intensive procedures, characterization over a wide age range is not feasible. In contrast, OCT can rapidly acquire in vivo retinal data, enabling more detailed characterization of age‐related changes. In cross‐sectional OCT studies, age has consistently been demonstrated to influence retinal thickness measurements in various layers (Girkin et al., 2011; Mwanza et al., 2011; Won et al., 2016), and the observed nonlinear patterns of change with decline beginning around the 30th decade are consistent with previous studies (Tong et al., 2019; Trinh et al., 2020; Yoshioka et al., 2017). While the varying regression models between different clusters within the GCL, INL, and ISOS suggested that absolute rates of change were variable across the macula, the consistency in percentage rates of change across the macula suggests that this does not reflect “true” variations in age‐related change with eccentricity, but are related to retinal thicknesses at a given location.

While longitudinal studies over extended years of observation are limited due to practical limitations in observing retinal changes over the wide age ranges per cross‐sectional studies, longitudinal studies of age‐related change are still important to consider, especially as imaging the same participants over time may provide more accurate characterizations of normal aging. Hammel et al. (2017) reported an average rate of change across the macular GC‐inner plexiform layer of −.14 μm/year, while averaged rates of change between −.25 and −.53 μm/year have been reported in the macular GC complex (Holló & Zhou, 2016; Zhang et al., 2016). While consistently slower than longitudinal changes in glaucoma eyes (Hammel et al., 2017; Holló & Zhou, 2016), the varying rates of change in healthy eyes may reflect differences in the studied inner retinal complexes or ages investigated; while age ranges of healthy participants were not reported across these studies, Zhang et al. (2016) included participants up to 85 years of age, and the steeper slope of decline reported by this study may be consistent with more rapid change in the GCL indicated by quadratic regression models in the present study. Meanwhile, longitudinal studies have reported no change in INL volume in healthy controls, patients with multiple sclerosis or neuromyelitis optica (Balk et al., 2019; Oertel et al., 2018); however, given the median durations of follow‐up in these studies were 2.0–2.3 years and the relatively flat quadratic regression models for the INL described in the present study, it is possible that the length of follow‐up was insufficient to observe decline in the INL. Longitudinal studies following retinal layer thicknesses over longer durations of follow‐up and including a larger range of ages would be valuable to confirm the findings of the present study.

### 4.2 Demographic factors influencing retinal thicknesses

Previous studies have variably reported reduced GCL thickness with increasing myopia and no effect with refractive error (Mwanza et al., 2011; Omoto et al., 2020; Sezgin Akcay et al., 2017; Tong et al., 2020), and a single study investigating the INL reported significant reductions in high myopia (Kim et al., 2020). The apparent reduction with increasing myopia may be related to transverse magnification effects rather than indicative of “true” reductions in retinal thickness with myopia (Lal et al., 2021; Lee et al., 2021; Omoto et al., 2020). As this study applied relatively constrained refractive error criteria, corrections for transverse magnification may be more pertinent for highly myopic eyes. Similarly, this study found no significant relationship between ethnicity and GCL thickness, consistent with Perez et al. (2021); however, other studies have reported significant differences in inner retinal thickness between different ethnicities (Khawaja et al., 2020; Poh et al., 2020), perhaps due to various ethnicities included across studies. In the present study, participants were only categorized as Asian or white, representing the patient demographic at Centre for Eye Health; a more ethnically diverse cohort may elucidate the relationship between ethnicity and inner retinal thicknesses in greater detail.

Our finding of a significant association between sex and INL thickness appears consistent with previous studies observing thinner INL measurements in women (Ooto et al., 2011; Won et al., 2016). Meanwhile, the present study did not observe a significant association between sex and ISOS thickness; however, previous studies have variably reported significantly thicker ISOS measurements in men and no sex‐dependent variations (Chua et al., 2019; Ooto et al., 2011; Won et al., 2016). Interestingly, Hermenean et al. (2020) observed differences in retinal microRNA expression between male and female mice correlating with histologically‐derived outer retinal thickness measurements, providing physiological mechanisms underlying sex‐related differences. Overall, these findings suggest that adjustment for sex may be required when investigating the INL, with possible ramifications in normative database comparisons for detection of pathology affecting the INL.

### 4.3 Clinical relevance of the INL and its cell population subtypes

Despite little literature on pathological variations in INL thickness, the various cellular constituents of the INL can be affected in ocular pathologies. For example, in diabetic retinopathy, microaneurysms and macular edema are primarily located in the INL (Chen et al., 2022; Das et al., 2018; Tsuboi et al., 2021), and INL thickness and perfusion have been significantly correlated with functional outputs such as visual acuity and visual field sensitivity (Mokrane et al., 2021; Tsuboi et al., 2021). Meanwhile, ex vivo studies of human and rodent retinas have revealed changes to the morphology and protein expression of Müller cells and amacrine cells (Gastinger et al., 2006; Hammes et al., 1995; Mizutani et al., 1998), and bipolar cell dysfunction has been observed as a consequence of GC disease and photoreceptor degeneration (Kosta et al., 2021; Shen et al., 2019). Moreover, cone bipolar cell loss and dysfunction in neurochemical signaling has been reported in rat models of retinal ischemia and reperfusion, with reductions in OCT‐derived INL thickness in human eyes with retinal ischemia appearing to reflect these changes (Kalloniatis et al., 2022; Sun et al., 2007). These suggest that structural changes in the INL may be useful biomarkers for various ocular pathologies. While the present study has derived densities of the various cell types residing in the INL based on averaged histological proportions from normal retinas, given inadequate resolution afforded by spectral‐domain OCT to visualize individual INL cell types, it would be ideal to confirm this study's findings with more advanced technologies. For example, visible‐light OCT imaging enabling visualization of INL sublayers may enable individualized cell density measurements based on substrata thickness measurements (Ghassabi et al., 2022). As various pathologies may cause alterations to specific INL cell subtypes, technologies enabling the visualization of changes to INL sublayers would have potential to improve detection of INL cell loss.

### 4.4 Combined role of OCT and emerging imaging technologies

While the distributions of OCT‐derived retinal thickness measurements appear to correspond to histological distributions of corresponding retinal cells, retinal thickness measurements do not necessarily consider eccentricity‐dependent variations of cell populations residing within the same layer. A classic example includes the distribution of rods and cones, with the peak in cone density at the foveal center and peak rod density reached at 1.2–1.7 mm eccentricity (Curcio et al., 1990; Scoles et al., 2014). This difference, in conjunction with possible effects of age‐ and sex‐distribution differences between historical histological and this study's OCT cohorts, is likely to contribute at least in part to apparent discrepancies between age‐related decline in OCT‐derived ISOS thickness, which was −.06% in this study, compared to an equivalent annual reduction of −0.54% a year in rod density as described by Curcio et al. (1993). Similarly, the distributions of rod and cone ON bipolar cells generally follow this eccentricity‐dependent trend, with more rod bipolar cells located more peripherally (Lee et al., 2019; Masri et al., 2021). As such, the relative contribution of each co‐stratified cell type to a given retinal thickness measurement can only be estimated based on proportional surface area calculated from cell densities and diameters.

The development of adaptive optics‐OCT has enabled the resolution of numerous retinal cell types (Liu et al., 2017; Rossi et al., 2017; Scoles et al., 2014; Wells‐Gray et al., 2016), and combining the identification of different cell types en face and retinal thicknesses may provide more precise, individualized estimates of cell densities. Moreover, adaptive optics studies have observed enlargement of GC somas in glaucoma (Liu et al., 2021; Soltanian‐Zadeh et al., 2021), suggesting that modifications to cell densities calculated from GCL thickness changes may be required to obtain more precise cell calculations. While the investigations in the present study provide a useful foundation for calculating cell densities from OCT, further work on cell morphology changes in disease would be highly beneficial to determine appropriateness in disease settings.

### 4.5 Limitations

In addition to those identified before, a key limitation of this study's design is its cross‐sectional comparison of histological and OCT data from different human cohorts, including slight differences in age and sex distributions between them due to the limited sample size of the histological cohorts affecting more direct matching of participants. While a longitudinal study design following a reasonably large human sample, including histological and OCT data, would be ideal, the ex vivo methods required to process histological data and the large age range required to sufficiently characterize change over an adult human's expected lifetime limits the practicality of such a longitudinal study design. Moreover, the comparability of corrected and age‐similar retinal thickness measurements in our study indicates that the derived regression models appear to characterize age‐related change appropriately. Another limitation includes reliance on human histological studies, generally including few participants from a limited age range, so cell density data obtained from these studies may not be entirely reflective of the spectrum of normative cell densities. GC and photoreceptor cell densities were also only available along principal meridians, and INL cell densities were only available along the temporal meridian, resulting in assumptions of linear change between meridians and similar densities regardless of angular eccentricity respectively. Furthermore, calculations of rod diameter performed in this study were based on assumptions of cone and rod density and packing and appeared to overestimate rod diameter centrally compared to the limited information on human rod diameter (Jonas et al., 1992). Lastly, as photoreceptor outer segments reportedly orient toward the nodal point of the human eye (Enoch, 1972; Laties & Enoch, 1971), there may be small mismatches between retinal thickness measurements extracted perpendicular to B‐scan tilt, as performed in this study, and histological photoreceptor thicknesses. Further detailed characterizations of quantitative cell density and morphological parameters with eccentricity and between principal meridians, perhaps with adaptive optics OCT, may help overcome these concerns in future work.

## 5 CONCLUSION

This study sought to develop a high spatial density OCT‐based model of the neural retinal layers, with appropriate correction for age and sex, to enable the calculation of cell density parameters from OCT. With correction factors derived from these models, the resultant minimal inter‐cohort variability indicates that OCT retinal thicknesses could be applied as surrogate structural parameters to cell densities derived from histological studies. Furthermore, the derivation of volumetric cell density, based on histological cell densities and retinal thickness measurements, allows for the calculation of retinal cell density from live human eyes with relative ease, in turn enabling greater translation of knowledge of cellular processes to clinical and research investigations.

## AUTHOR CONTRIBUTIONS

All authors have contributed to the manuscript substantially and have agreed to the final submitted version.

## CONFLICT OF INTEREST STATEMENT

The authors have no conflict of interests to declare.

## TRANSPARENT PEER REVIEW

The peer review history for this article is available at https://publons.com/publon/10.1002/cne.25483.

## Supporting information

Supplementary Table

## Data Availability

The data that support the findings of this study are available in the Supporting Section of this article.
